# Hardy exotics species in temperate zone: can “warm water” crayfish invaders establish regardless of low temperatures?

**DOI:** 10.1038/srep16340

**Published:** 2015-11-17

**Authors:** Lukáš Veselý, Miloš Buřič, Antonín Kouba

**Affiliations:** 1 University of South Bohemia in České Budějovice, Faculty of Fishery and Protection of Waters, South Bohemian Research Centre of Aquaculture and Biodiversity of Hydrocenoses, Zátiší 728/II, 389 25 Vodňany, Czech Republic

## Abstract

The spreading of new crayfish species poses a serious risk for freshwater ecosystems; because they are omnivores they influence more than one level in the trophic chain and they represent a significant part of the benthic biomass. Both the environmental change through global warming and the expansion of the pet trade increase the possibilities of their spreading. We investigated the potential of four “warm water” highly invasive crayfish species to overwinter in the temperate zone, so as to predict whether these species pose a risk for European freshwaters. We used 15 specimens of each of the following species: the red swamp crayfish (*Procambarus clarkii*), the marbled crayfish (*Procambarus fallax* f. *virginalis*), the yabby (*Cherax destructor*), and the redclaw (*Cherax quadricarinatus*). Specimens were acclimatized and kept for 6.5 months at temperatures simulating the winter temperature regime of European temperate zone lentic ecosystems. We conclude that the red swamp crayfish, marbled crayfish and yabby have the ability to withstand low winter temperatures relevant for lentic habitats in the European temperate zone, making them a serious invasive threat to freshwater ecosystems.

Invasive species are one of the most serious threats to global biodiversity^1^,^2^. The introduction of exotic species outside of their native range may have far-reaching ecological consequences on local communities as well as causing unpredictable impacts, even for humans^3^,^4^,^5^. Most non-indigenous species have had minimal negative effects and they usually fail to become established, or if they become naturalized, they constitute only a small portion of the community with no significant effects^6^,^7^. However, some of them are able to adapt to new conditions^6^ and strongly affect the ecosystems they invade^8^.

Crayfish may represent up to 85% of the local zoobenthic biomass^9^ and they are considered as keystone species^3^,^10^; they are strong ecosystem engineers, modifying the environment to suit themselves^11^,^12^. Based on previous studies, the introduction of non-indigenous crayfish species have caused major and almost irreversible changes in native freshwater crayfish stocks^13^,^14^,^15^. In addition to imposing strong competitive pressures on the native crayfish populations, they also may alter entire ecosystems and their food webs^13^,^14^. Red swamp crayfish, *Procambarus clarkii,* has been well studied and their negative impact is often cited as an example^16^,^17^. Although not as well studied, other alien crayfish species , e.g., the marbled crayfish *Procambarus fallax* f. *virginalis,* yabby *Cherax destructor* and the redclaw *Cherax quadricarinatus* etc. are considered to cause detrimental impacts once established in the natural environment^8^, these new invaders warrant more attention relative to potential regulation or management.

The aquarium trade is considered to be one of the main pathways for introduction^18^. As all four species are regarded as “warm water”, global warming may increase their potential to become established in previously unavailable or unsuitable habitats^19^,^20^. The ability of non-indigenous species to withstand temperatures outside their natural optima (e.g., low winter temperature in the temperate zone) is a necessary prerequisite for survival and spread. Several studies have been carried out in Europe on the spread and reproduction of invasive poikilothermic organisms^21^,^22^, but only a few concerning crayfish^19^,^20^,^23^. However, while the distribution models based on laboratory studies are not fully predictive, they provide evidence that European conditions might already be suitable for warm water crayfish with further increasing suitability in the future.

We focused on four non-European crayfish species that can be considered as “warm water” from the Central European perspective; their recent geographic distribution supports this view^24^. The red swamp crayfish is native to the southeastern United States and Mexico^25^,^26^. Its European occurrence is concentrated in to the southwestern part of the continent, having invaded at least 15 territories ranging from Spain to Germany^24^. The native range of the marbled crayfish is unknown; however, peninsular Florida and southern Georgia have been suggested, due to its close affinity to *Procambarus fallax* originating from that region^27^. Records in Europe are rapidly increasing, with reports of populations having been established in Germany and Slovakia^24^,^28^; thus, indicating the potential to spread to other European countries. The yabby, *Cherax destructor,* is the most widespread crayfish species in Australia; it is native to semiarid and arid parts of the continent^29^. Its original distribution was southern and eastern Australia (the states of New South Wales, Victoria^30^, but it may also be found in Queensland and a part of the Northern Territory^31^). The redclaw, *Cherax quadricarinatus,* is native to the northern part of Australia and to southeastern New Guinea^32^. In Europe, these Australian crayfishes so far have a limited distribution (Spain, Italy, and Slovenia) where the redclaw inhabits an oxbow lake with an elevated temperature due to thermal springs^24^,^33^. All these species are widely kept as pets, both in Europe and North America^34^,^35^,^36^ and all but one (marbled crayfish) are used in aquaculture. Animal escapes or releases have been already proved to be the most likely sources of introductions in several crayfish species^24^. In this study, we tested the overwintering capability of the four species so as to predict the potential risk of their establishment in the freshwater ecosystems of temperate Europe.

## Results

**Figure 1:**
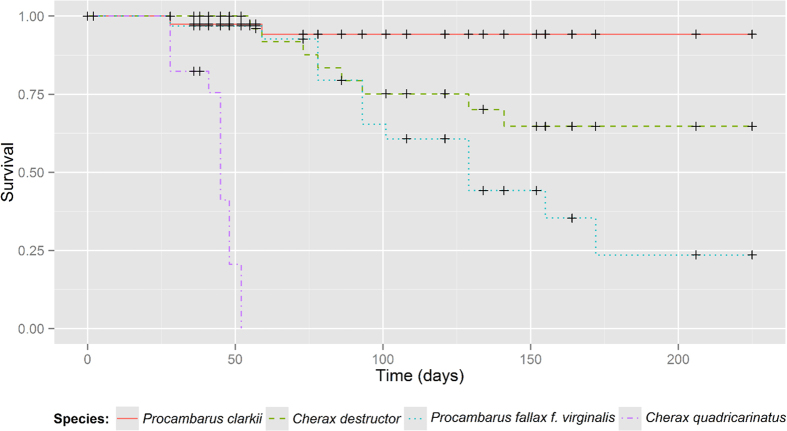
Survival analysis plot of chosen non-indigenous crayfish.

Marked differences in survival were observed among the tested species (χ^2^ = 136, df = 3, p = <0.001; Fig. 1; see Table S1). We found that the red swamp crayfish was the most capable of withstanding the experimental conditions; with 13 out of 15 surviving specimens. Moderate resistance was found in yabby, where 7 out of 15 animals survived. All the mortality occurred during the exposure to the hardest winter conditions (2–3 °C; days 55–141). Similarly, ten marbled crayfish died between 78 to 129 days when the temperature was the lowest. Only one marbled crayfish survived the whole experimental trial. The lowest survival was recorded in redclaw, demonstrating they were the least tolerant of the low temperature conditions; all specimens died between days 41 to 52 when the temperature declined to 2-3 °C. Survival was not related to size; no significant correlation was found between crayfish winter survival and their weight, carapace or postorbital lengths (One-way ANOVA p = 0.11–0.81).

**Figure 2:**
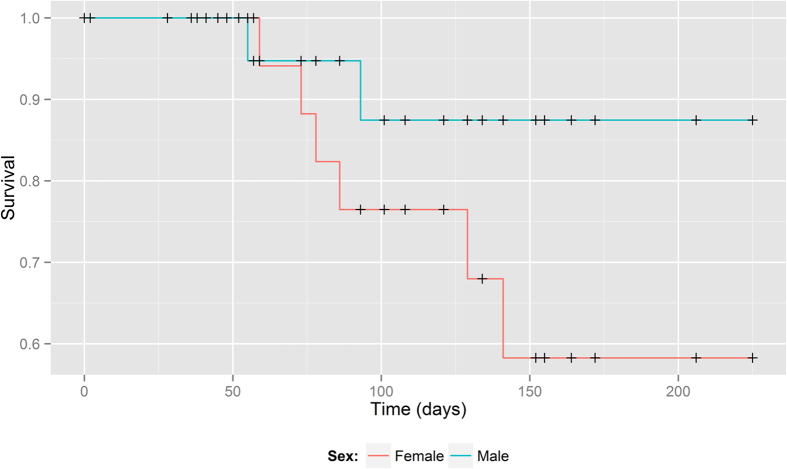
Survival analysis plot of *Cherax destructor*.

Differences in survival were found in males and females of yabby (χ^2^ = 2.9, df = 1, p = 0.048; Fig. 2; see Table S2). Individuals of both sexes withstood the experimental conditions; however, males survived at a higher rate. No such differences were observed for redclaw (χ^2^ = 0.3, df = 1, p = 0.61) and red swamp crayfish (χ^2^ = 0.1, df = 1, p = 0.80).

**Figure 3:**
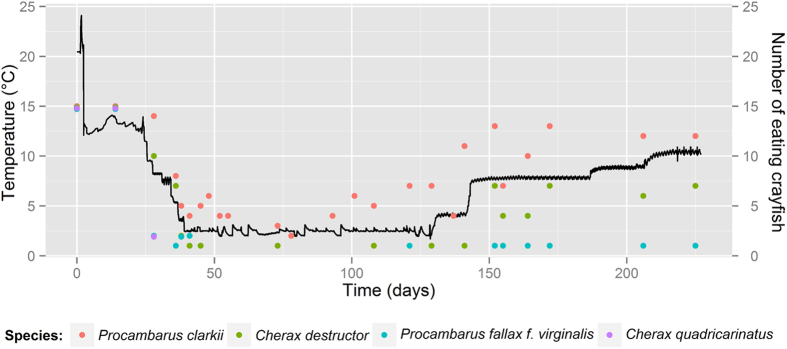
Temperature and foraging activity of chosen non-indigenous species during the experiment.

Foraging activity of crayfish was also recorded during the experiment (Fig. 3). We observed that at least two red swamp crayfish were feeding even during the coldest period, but generally around four specimens fed at this time. Numbers of feeding crayfish rose substantially with increasing temperature. In case of the yabby, food intake decreased during acclimatization. Finally, only one specimen irregularly ate during the coldest period. Marbled crayfish almost did not eat during coldest period. In fact, only the one surviving specimen occasionally ate. Feeding activity was low in the redclaw and completely stopped after 28 days i.e. during acclimatization.

## Discussion

Catford *et al.*^37^ suggests that, after propagule pressure the first set of factors that must be met for species establishment are abiotic conditions, importantly including temperature and other environmental conditions. In this study we examined one of most important factors that influences or determines the freshwater biota composition. We found that three out of four species survived simulated winter conditions (Fig. 1; see Table S1). Red swamp crayfish had the highest survival rate and foraging activity, followed by yabby and marbled crayfish. Redclaw suffered high mortalities and did not survive the lowest temperatures.

Both a distribution model^19^ and use of “avatar” species^38^ suggested Europe could be a suitable place for red swamp crayfish establishment. Similarly, our results revealed winter mortality is exceptionally low compared to the other tested species. Red swamp crayfish were able to withstand the coldest period with a temperature of around 2.5 °C, similar to the one occurring in waterbodies that freeze during the winter, where its survival has already been documented^30^. In the central part of Europe, populations are established in Austria and Germany. However, the Austrian occurrence is represented by a single population settled in a thermal stream^39^. Chucholl^40^ describes the life history of this species in new cold habitats situated in southern Germany and highlights its high plasticity. Unfortunately, no exact data about the course of winter temperatures are mentioned. Thus, our study provides interesting insight into red swamp crayfish temperature resistance in this respect. The red swamp crayfish could be a real problem for European temperate zone ecosystems as exemplified by substantial damage caused to local freshwater communities in southwestern Europe^41^. This non-indigenous species exhibits extended environmental resistance, substantial plasticity, extreme aggressiveness and high population densities that favor the displacement of native European crayfish^42^,^43^,^44^. In addition, its ability to resort to riparian and terrestrial feeding when the freshwater sources are depleted has been documented^17^. Moreover, it is also capable of transmitting the crayfish plague pathogen to native European crayfish species^45^.

With respect to winter survival it is also necessary to mention that there is more than one factor determining population success. For example, the temperature during the whole season is also important. If the temperature during the season is not warm enough for egg development (degree days), the species also would fail to become established^46^. According to Suko^47^ red swamp crayfish demonstrate a link between warmer water temperatures and increased reproductive success in terms of multiple reproduction. It is partly caused by faster egg development in warmer temperature^48^. However, not only this species could represent this trends. All species involved in the trial are considered as warm water species and this trend could be highly expected among them.

Observed foraging activity in the coldest period is also surprising. Croll & Watts^49^ studying the effect of temperature on feed consumption and nutrient absorption reported that the red swamp crayfish were generally lethargic at 8 °C. Food consumption was minimal and remained low even at 14 °C compared to southern white river crayfish (*Procambarus zonangulus*). Populations of the latter species and some closely related taxa are also present in European open waters^24^,^50^. Winter food intake, likely occurring in both aforementioned species, might have negative impacts on local communities although it could be much reduced by the low temperature.

We also found that one red swamp crayfish male, at the end of acclimatization period when the temperature reach 4 °C, molted. It did not eat its exuvium and its carapace remained soft but the individual crayfish survived until the end of the experiment. Although its survival in natural conditions may be difficult as an uncalcified crayfish will be an easy prey for potential predators, it still illustrates the substantial physiological abilities of the species.

Considering the native range and recent distribution of yabby in Europe, covering Spain and Italy^24^, the discovery its substantial resistance to experimental conditions is surprising. Although winter mortality was higher compared to red swamp crayfish, the yabby still exhibited a good ability to survive. According to Capinha *et al.*^51^ to date, yabby could occupy nine times higher suitability areas outside the species current ranges. On the other hand it is predicted to have lower climatic suitability in Italy^52^ and with a view to future Spain seems to be less suitable for yabby in terms of climatic change^19^. Moreover according to model prediction the mean annual temperature and mean temperature of the coldest quarter seems to be the best predictor of future distribution^53^.

Considering our results, we expect that the establishment of new populations of yabby in the temperate zone of Europe is highly possible. The female’s mortality was higher compared to the male. On the other hand, winter conditions pose a new challenge for a warm water species and the survivors’ sex ratio could be changed due to a different life strategy in term of multiple reproductions in one season and the related higher energy and nutrient demands in females. Still, the number of female survivors could be sufficient to establish a new population.

According to Withnall^31^ “*Cherax destructor* is adapted to a wide range of water temperatures, between 1 °C and 35 °C. It does not grow at water temperatures below 15 °C and falls into a state of partial hibernation (i.e. metabolism and feeding cease) when water temperature drops below 16 °C”. These results are in contrast to ours, where foraging activities were observed even in the coldest period, although a certain degree of variability among the studies is expected. Finding the substantial resistance of yabby to low water temperatures opens an array of topics, including assessment of its life history at both recently known and possibly new localities, and presumed impacts on European freshwater communities.

Marbled crayfish suffered massive mortality and only a single specimen survived in our experiment. However, due to its parthenogenetic reproduction strategy, theoretically no more than one individual is needed to establish a viable population and potential invasiveness is therefore extremely high^54^,^55^. The parthenogenetic reproduction strategy combined with fast growth, early maturation, high fecundity, and a capacity for competing with other crayfish species^56^,^57^ provides this crayfish species an enhanced chance of success. Marbled crayfish are already present and flourish in Germany^58^, Slovakia^59^ and likely also in other European states e.g., Italy^60^ and Croatia^61^ and future spread throughout the Europe is predicted^28^.

Redclaw will clearly not be a threat to European temperate zone ecosystems in the near future which is in accordance with the distribution model of Larson and Olden^38^. The likelihood of its establishment in the southwestern part of Europe may increase along with the temperature rise caused by a global warming, unless the region is fully occupied by species carrying the infectious mold *Aphanomyces astaci*. In this context, also the presence and competition with freshwater crab species might act as a limiting factor^62^,^63^. According to previous study from Israel where survival of redclaw was tested in earthen ponds during the winter, 60% of specimens survived^64^. Such climatic conditions are generally comparable to Mediterranean European winters, suggesting the ability of redclaw to survive in this region and consequently spread as far as the resistance to the low temperatures is considered.

## Conclusion

To sum up, all the species mentioned are widely traded as pets in Europe and North America^34^,^35^,^36^, which might facilitate their further spread throughout Europe^24^. Only red swamp crayfish demonstrated high survival in experimental conditions and exhibited feeding activity even during the coldest period. Thus, this species could be regarded as a major threat to lentic freshwater habitats in Central Europe. Considering the native range and recent distribution of the yabby in Europe, its ability to withstand low temperatures indicates a potential to spread and should highlight the need for consideration of this species’ environmental plasticity. Finally, our results clearly indicate that the redclaw will not pose a risk for European temperate zone.

## Methods

Given current condition the study was conducted at the Research Institute of Fish Culture and Hydrobiology in Vodňany, Czech Republic, from the second half of December 2013 to the first half of July 2014. No specific permissions were required for the locations and activities involved in this study. The study did not involve endangered or protected species. All experimental manipulations (rearing, capture and measurements) were conducted according to the principles of the Institutional Animal Care and Use Committee (IACUC) of the University of South Bohemia, Faculty of Fisheries and Protection of Waters, Research Institute of Fish Culture and Hydrobiology, Vodňany, based on the EU harmonized animal welfare act of Czech Republic. The above mentioned Ethical Committee (IACUC) specifically approved this study. The principles of laboratory animal care and the national laws 246/1992 and regulations on animal welfare were followed (Ref. number 22761/2009-17210).

Crayfish were placed individually into one-liter plastic boxes which was placed into the temperature regulated incubator. Half of the box, as well as lid, were covered with black tape to provide shelter. The lids were perforated (10 holes per lid with size 0.5 cm^2^ per hole) to ensure air exchange; winter temperature and light regimen were set according to lentic conditions in temperate continental Europe. The temperature was recorded at one hour intervals by a datalogger MINIKIN (Environmental measuring systems, Brno, Czech Republic). Crayfish were fed during each control (see below) with a slice of carrot and also, once a month, with worms (*Tubifex tubifex*); unconsumed food was removed. Crayfish feeding, surviving and molting were recorded. Absolute food consumption was not recorded, only its occurrence.

### Experimental design

Fifteen adult specimens of each of the four non-indigenous crayfish species were used (Table 1). The sexes were generally equally selected with exception that the red swamp crayfish had more males (12 males vs. 3 females) and only females were used for the marbled crayfish. Each specimen was measured and weighed to the nearest 0.1 mm and 0.1 g, respectively.

The 226-day experiment was divided into three parts. During the initial 40-d acclimatization period, the temperature was decreased from 25 to 3 °C. In the second part, winter lentic conditions were simulated by maintaining temperature at 2–3 °C for 90 days. During the final two months spring conditions were simulated by gradually increasing temperature from 2–3 °C to 10 °C. In the first three months crayfish were checked once per week because of the predicted high mortality. After this period, the animals were checked each 14 days till the end of the experiment. In order to maintain water quality, water was changed by tempered tap water during the controls. Water quality parameters of water before exchange (n = 2) were as follows: ANC_4.5_ (acid neutralization capacity)  =  1.0 ± 0.1 mmol·l^−1^; ammonia nitrogen 0.2 ± 0.0 mg·l^−1^; nitrites < 5.00 mg·l^−1^; nitrates 0.03 ± 0.0 mg·l^−1^; calcium 24.4 ± 1.9 mg·l^−1^; phosphorus 0.1 ± 0.0 mg·l^−1^; magnesium 2.4 ± 0.2 mg·l^−1^.

### Statistical analysis

For statistical evaluation of the dataset we used the R-statistics software with packages: survival^65^, Kmsurv^66^, GGally^67^ and ggplot2^68^. Non-parametric survival analysis (Kaplan-Meier method) was performed for all species as well as for both sexes in each species (if applicable). One-way ANOVA was performed to test whether length of carapace or weight of specimen influenced survival during the winter.

## Additional Information

**How to cite this article**: Veselý, L. *et al.* Hardy exotics species in temperate zone: can “warm water” crayfish invaders establish regardless of low temperatures? *Sci. Rep.* **5**, 16340; doi: 10.1038/srep16340 (2015).

## Supplementary Material

Supplementary Information

## Figures and Tables

**Table 1:** Mean size expressed as carapace length (CL) and weight (W) of chosen species.

	CL (mm)	W (g)
*Procambarus clarkii*	32.3 ± 3.1	9.1 ± 2.0
*Procambarus fallax* f. *virginalis*	24.0 ± 2.7	4.4 ± 1.4
*Cherax destructor*	28.7 ± 2.7	8.5 ± 3.2
*Cherax quadricarinatus*	34.8 ± 5.6	9.5 ± 4.4
