# Supplementary material for: Hardy exotics species in temperate zone: can “warm water” crayfish invaders establish regardless of low temperatures?
Source: Sci Rep. 2015 Nov 17;5:16340. doi: 10.1038/srep16340 (PMC4648075; doi:10.1038/srep16340)
Supplement: Supplementary Information [file srep16340-s1.doc]

**Hardy exotics species in temperate zone: can “warm water” crayfish invaders establish regardless of low temperatures?**

Lukáš Veselý, Miloš Buřič, Antonín Kouba

University of South Bohemia in České Budějovice, Faculty of Fishery and Protection of Waters, South Bohemian Research Centre of Aquaculture and Biodiversity of Hydrocenoses, Zátiší 728/II, 389 25 Vodňany, Czech Republic

**Table S1 Survival analysis result of chosen non-indigenous crayfish species.**

Time – time in days when dead specimen was found, Number of event – the number of specimens found dead during given control.

| **Time (Days)** | **The number of survivors** | **Number of event** | **Survival** | **Standard error** |
| --- | --- | --- | --- | --- |
| ***Procambarus clarkii*** | | | | |
| 28 | 14 | 1 | 0.974 | 0.025 |
| 59 | 13 | 1 | 0.942 | 0.04 |
| ***Cherax destructor*** | | | | |
| 55 | 14 | 1 | 0.960 | 0.039 |
| 59 | 13 | 1 | 0.918 | 0.055 |
| 73 | 12 | 1 | 0.877 | 0.067 |
| 78 | 11 | 1 | 0.835 | 0.076 |
| 86 | 10 | 1 | 0.793 | 0.083 |
| 93 | 9 | 1 | 0.751 | 0.088 |
| 129 | 8 | 1 | 0.701 | 0.095 |
| 141 | 7 | 1 | 0.647 | 0.102 |
| ***Procambarus fallax* f. *virginalis*** | | | | |
| 28 | 14 | 1 | 0.969 | 0.031 |
| 59 | 13 | 1 | 0.927 | 0.051 |
| 78 | 12 | 3 | 0.794 | 0.083 |
| 93 | 9 | 3 | 0.654 | 0.1 |
| 101 | 6 | 1 | 0.607 | 0.103 |
| 129 | 5 | 3 | 0.442 | 0.111 |
| 155 | 2 | 1 | 0.353 | 0.119 |
| 172 | 1 | 1 | 0.236 | 0.125 |
| ***Cherax quadricarinatus*** | | | | |
| 28 | 12 | 3 | 0.824 | 0.093 |
| 41 | 9 | 1 | 0.755 | 0.107 |
| 45 | 8 | 5 | 0.412 | 0.128 |
| 48 | 3 | 3 | 0.206 | 0.106 |
| 52 | 0 | 3 | 0.000 | NaN |

NaN – not a number

**Table S2 Survival analysis result of male and female of yabby (*Cherax destructor)*.** Time – time in days when dead specimen was found, Number of event – the number of specimens found dead during given control.

| **Time (Days)** | **The number of survivors** | **Number of event** | **Survival** | **Standard error** |
| --- | --- | --- | --- | --- |
| **Female** | | | | |
| 59 | 7 | 1 | 0.941 | 0.057 |
| 73 | 6 | 1 | 0.882 | 0.078 |
| 78 | 5 | 1 | 0.824 | 0.092 |
| 86 | 4 | 1 | 0.765 | 0.103 |
| 129 | 3 | 1 | 0.680 | 0.122 |
| 141 | 2 | 1 | 0.583 | 0.138 |
| **Male** | | | | |
| 55 | 6 | 1 | 0.947 | 0.051 |
| 93 | 5 | 1 | 0.874 | 0.085 |
